# Clinical and microbiological features of *Actinotignum* bacteremia: a retrospective observational study of 57 cases

**DOI:** 10.1007/s10096-016-2862-y

**Published:** 2016-12-12

**Authors:** H. Pedersen, E. Senneby, M. Rasmussen

**Affiliations:** 1 Department of Clinical Sciences, Division of Infection Medicine, Lund University, BMC, B14, Tornavägen 10, 22184 Lund, Sweden; 2 Clinical Microbiology, Region Skåne, Lund, Sweden

## Abstract

The purpose of this study was to investigate the incidence, clinical presentation, and prognosis of *Actinotignum* bacteremia in southern Sweden. *Actinotignum* isolates in blood cultures were identified retrospectively between 1st January 2012 and 31st March 2016 through searches in the clinical microbiology laboratory database. The population covered by this laboratory is approximately 1.3 million. Matrix-assisted laser desorption/ionization time-of-flight mass spectrometry (MALDI-TOF MS) was used for species determination. Etests were used for minimum inhibitory concentration (MIC) determination. The patients’ medical charts were reviewed. Fifty-eight episodes in fifty-seven patients with *Actinotignum* bacteremia were identified (*A. schaalii* = 53, *A. sanguinis* = 1, *A. urinale* = 2, and *Actinotignum* species = 3), which corresponds to an incidence of 11 cases per million inhabitants. Fifty-one percent of the isolates were in pure culture. The MICs were low for β-lactam antibiotics, whereas high MICs were recorded for ciprofloxacin and trimethoprim. Patients had a median age of 82 years, 72% were male, and a majority had underlying urological conditions. Thirty-six of the patients were diagnosed with a focus from the urinary tract. Thirty-one patients developed severe sepsis and nine patients died during the hospital stay. Our study is the largest of *Actinotignum* bacteremia and demonstrates that it is a condition with a significant fatality that affects elderly persons with underlying conditions. β-Lactams represent a rational treatment option.

## Introduction

The genus *Actinobaculum* was first described in 1997, when it was separated from *Actinomyces* [1, 2]. Several species belonging to *Actinobaculum* were reclassified in 2015 and they were proposed to constitute a novel genus, *Actinotignum* [2]. This genus now comprises three species: *Actinotignum schaalii*, *Actinotignum sanguinis*, and *Actinotignum urinale* [2]. These bacteria are small facultative anaerobic Gram-positive rods that are non-motile and catalase-negative [1]. They grow slowly and preferably under anaerobic or CO_2_ conditions, and are easily overgrown by other bacteria [3]. *Actinotignum* species are probably part of the normal urogenital flora [4] and have been associated with urinary tract infections [5, 6]. They are, however, most likely under-diagnosed in urine cultures, since most clinical microbiological laboratories routinely only use aerobic growth conditions. Matrix-assisted laser desorption/ionization time-of-flight mass spectrometry (MALDI-TOF MS) appears to be a useful method for correct species determination of these bacteria [3].

*Actinotignum schaalii* has been associated with urinary tract infections [6, 7] but can also cause invasive infections, such as Fournier’s gangrene, urinary bladder necrosis, bacteremia, and endocarditis [8–11]. The characteristics of infections with *A. schaalii* have been described in two recent reviews [5, 12]. However, only a few studies have addressed the clinical presentation of *Actinotignum* bacteremia. In a study from Denmark, 98 patients with *A. schaalii* bacteremia were identified between 2011 and 2014, but the clinical presentation was only described for ten patients [8]. These had a median age of 79 years, 70% were male, and 50% had predisposing urogenital conditions. In a Swedish study, 17 cases of *A. schaalii* bacteremia were identified retrospectively and patients were shown to typically be older males with underlying urogenital risk factors [13]. Similar results have been indicated in a case series from Spain [14], as well as in reports of single cases and smaller case-series [7, 15–20]. Given the low number of cases reported, the prognosis of *Actinotignum* bacteremia is not known.

In published cases, the preferred treatment of *A. schaalii* bacteremia has been with β-lactams to which the bacterium has low minimum inhibitory concentration (MIC) values. However, since bacteremia typically has a urinary tract focus, there is a risk that empiric treatment will be given with antibiotics such as trimethoprim–sulfamethoxazole or ciprofloxacin, to which the bacterium is resistant in vitro [16, 19, 21]. The duration of the treatment is still uncertain but there have been failures after 1 week of ampicillin treatment [6, 19], which indicates that longer treatment periods may be needed. In this retrospective study, we present the incidence, clinical presentation, and prognosis of *Actinotignum* bacteremia in southern Sweden.

## Materials and methods

### Setting

Patients with any *Actinotignum* species in blood cultures between 1st January 2012 and 31st March 2016 were identified through searches in the database belonging to the clinical microbiology laboratories in Malmö and Lund, Sweden. This database contains clinical samples from all ten hospitals within Region Skåne, with 1,303,627 inhabitants in 2015 [22]. The laboratories used blood culture systems and MALDI-TOF MS, as previously described [23]. We performed species determination with 16S rRNA gene sequencing with fD1 mod and P911 primers, as previously described [24], on all isolates where both the initial and renewed MALDI-TOF MS analysis gave a score below 2.0.

### Clinical presentation

The medical charts were studied and the following variables were extracted: age, underlying diseases, urogenital risk factors, symptoms, vital signs, laboratory values, antibiotic treatment, diagnosis, outcome, and other microbiological findings. Urogenital risk factors were defined as one or more of catheterization, prostatic hyperplasia, prostatic cancer, bladder cancer, hydronephrosis, renal failure, urethral stricture, or kidney stones. Severe sepsis was defined as one or more of hypotension with a systolic blood pressure below 90 mmHg, hypoperfusion with plasma lactate of >3.3 mM, or organ dysfunction. Organ dysfunction, in turn, was defined as an increase in plasma creatinine by >44 μmol/l, saturation <86% at admission (<78% if the focus of infection was the lungs), platelets <100 × 10^9^/l or PK/INR >1.5, an acute confusion and/or bilirubin >45 μmol/l [25]. A need for ventilation support, inotropic support, or continuous hemofiltration was also regarded as proof of organ dysfunction.

### Antibiotic susceptibility

Isolates were cultured for 48 h under anaerobic conditions (Whitley A35 Anaerobic Workstation) on blood agar plates [produced by the Clinical Microbiology Laboratory, Lund, Sweden, using Blood Agar Base LAB028 (LabM, Lancashire, UK), with the addition of 5% horse blood]. The MIC was determined using the Etest (bioMérieux, Marcy-l’Etoile, France) on Mueller–Hinton fastidious agar plates according to the instructions give by the manufacturer. The MIC was recorded after 24 or 48 h of incubation anaerobically.

### Statistical methods

Continuous variables were compared using the Mann–Whitney *U*-test and categorical variables using the Fisher’s exact test in Prism 7 for Mac OS X.

## Results

### Microbiology

A total of 58 episodes of *Actinotignum* bacteremia were identified, corresponding to an incidence of 11 cases per million inhabitants per year. Fourteen of the isolates had an MALDI-TOF MS score below 2.0 and were subjected to sequencing of the 16S rRNA gene for species determination. Eleven isolates were identified as *A. schaalii*, whereas three of the isolates could not be securely identified to the species level and were, thus, designated as *Actinotignum* species. *Actinotignum schaalii* was isolated from 53 patients, *A. sanguinis* was isolated from one patient, and *A. urinale* was isolated from two patients (once with *A. schaalii* and once with *Peptoniphilus harei*). One patient had a recurrent *A. schaalii* infection, with six months between the episodes, and only the first episode is reported here. In total, *Actinotignum* was the only finding in blood in 29 cases. In the other cases, additional bacterial species were isolated from the blood. The most common findings were *Aerococcus* species (*n* = 9), *Peptoniphilus* species (*n* = 4), *Escherichia coli* (*n* = 3), *Enterococcus faecalis* (*n* = 3), and *Proteus mirabilis* (*n* = 3). In four samples, more than two bacterial species of bacteria were isolated. The most common bacteria found in urine were *E. coli* (*n* = 14), *E. faecalis* (*n* = 5), and *Aerococcus* species (*n* = 3). Only one urine sample grew *A. schaalii*.

Of the 54 isolates available for antimicrobial susceptibility testing, all showed low MIC values to β-lactams and vancomycin, whereas the MIC values varied for erythromycin, clindamycin, and gentamicin. High MICs were recorded for trimethoprim and ciprofloxacin. The results are summarized in Table 1. European Committee on Antimicrobial Susceptibility Testing (EUCAST) breakpoints for Gram-positive anaerobes were used for classification of sensitivity [26].

**Table 1:** Minimum inhibitory concentration (MIC) values for the 54 *Actinotignum* isolates

Antibiotics	MIC values																	
	0.002	0.004	0.008	0.016	0.032	0.064	0.125	0.25	0.5	1	2	4	8	16	32	64	128	>256
Bensylpenicillin		2	9	26	14	3												
Ampicillin				4	2	2	6	15	16	5	4							
Cefotaxime			12	15	16	4	6	1										
Imipenem	1	3	16	23	10		1											
Vancomycin						1	19	31	2		1							
Gentamicin									2	15	32	1		3		1		
Ciprofloxacin									1	1	4	4	3	3	38			
Trimethoprim															54			
Clindamycin				19	16	10	1	1	1									6
Erythromycin				20	17	10			1									6

### Clinical presentation

The median age of the patients was 82 years and 72% were men. Only one of the patients had no underlying conditions. A majority of the patients (61%) had one or more underlying urogenital conditions, the most common being prostatic disorders (*n* = 16) and urinary catheter (*n* = 14). The patient characteristics are summarized in Table 2. The most common symptom at presentation was fever (61%). Nine of the patients presented with symptoms from the urinary tract, such as hematuria or strong smelling urine, and nine presented with unspecified abdominal pains. In eight subjects, the positive blood culture was drawn after more than 48 h of hospitalization and, thus, represent nosocomial infections.

**Table 2:** Clinical features of *Actinotignum* bacteremia

Demographics	
Median age (years)	82 (range 48–98)
Male sex	41 (72%)
Microbiology	
Pure culture	29 (51%)
Positive urine cultures	26 (46%)
Underlying conditions	
Urogenital conditions^a^	35 (61%)
Cardiovascular disease^b^	39 (68%)
Dementia	8 (14%)
Diabetes mellitus	11 (19%)
Symptoms and signs	
Fever	35 (61%)
Urinary tract symptoms	9 (16%)
Respiratory symptoms	17 (30%)
Severe sepsis	31 (54%)
Median CRP^c^ at hospitalization	59 mg/L (range 2–420)
Median WBC^d^ at hospitalization	14 × 10^9^/L (range 5.8–50)
Focus of infection	
Urinary tract	36 (63%)
Unknown	19 (33%)
Other	2 (4%)
Management and outcome	
Initial antibiotic effective against *Actinotignum*	53 (93%)
Median in-hospital time (days)	10 (range 2–25)
Echocardiogram	8 (14%)
ICU^e^	5 (9%)
Deceased during hospitalization	9 (16%)

^a^Underlying conditions defined as one or more of catheterization, prostatic hyperplasia, prostatic cancer, bladder cancer, hydronephrosis, renal failure, urethral stricture, lower urinary tract symptoms (LUTS), or kidney stones

^b^Underlying conditions defined as atrial fibrillation, pacemaker, ischemic heart disease, myocardial infarction, or stroke

^c^C-reactive protein

^d^White blood cells

^e^Intensive care unit

### Course and outcome

Thirty-one patients developed severe sepsis, and in 39% of these cases, more than one organ failure was recorded. Thirteen of the patients with severe sepsis had *Actinotignum* as the single organism isolated from blood, whereas 18 of these patients had polymicrobial bacteremia. Renal failure was the most common organ failure (*n* = 17), followed by hypoperfusion (*n* = 10) and respiratory failure (*n* = 8). Five patients were treated in the intensive care unit with non-invasive ventilation or vasopressor support. Thirty-six patients were diagnosed with a focus in the urinary tract and 11 with pneumonia, though chest radiograms were not conclusive for pneumonia in any of these cases. Eight patients were subjected to echocardiography, which did not reveal signs of endocarditis. Fifty-three patients received adequate antibiotic treatment initially, of which 43 were treated with β-lactams. The most common follow-up treatment was per oral amoxicillin (*n* = 19). A few patients received ciprofloxacin as follow-up. The median time of the total antibiotic treatment was 14 days. The median time of hospitalization was 10 days for survivors. Nine of the patients died during hospitalization. The features of deceased patients and survivors are given in Table 3.

**Table 3:** Features of survivors and the deceased

	Survivors	Deceased	*p*-Value
Median age	81 years	86 years	0.03
Male sex	33/48 (69%)	8/9 (89%)	0.4
Pure culture	26/48 (54%)	2/9 (22%)	0.1
Severe sepsis	22/48 (46%)	9/9 (100%)	0.003
Median CRP^a^ at hospitalization	58	59	0.3
Median WBC^b^ at hospitalization	13	16	0.3
Underlying urogenital conditions	29/48 (60%)	5/9 (56%)	1
Urinary tract focus of infection	32/48 (67%)	3/9 (33%)	0.1
Nosocomial infection	5/48 (10%)	2/9 (22%)	0.3
Adequate initial antibiotic treatment	44/48	9/9	1

^a^C-reactive protein

^b^White blood cells

## Discussion

The number of reported cases of *Actinobaculum*/*Actinotignum* bacteremia has been very low, possibly due to the relatively recent identification of the species and missed identification in the microbiological laboratories. With MALDI-TOF MS as the primary species identification method and an increasing number of older persons with urinary tract morbidity, *Actinotignum* infections will likely be more frequently encountered. We report an incidence of *Actinotignum* bacteremia of 11 cases per million inhabitants per year, which is higher than what is suggested by a recent Danish study [8]. Our results confirm previous studies showing that patients affected by the condition are typically older males with underlying urinary tract conditions [8, 13, 14]. From our case series, which is the largest one to date, the prognosis of *Actinotignum* bacteremia can be assessed. We report a relatively high mortality (16%), which is likely related to the advanced age and co-morbidity of the patients. Some of the deceased patients had polymicrobial bacteremia and the exact contribution of *A. schaalii* to the outcome is difficult to determine.

*Actinotignum schaalii* is the most commonly isolated *Actinotignum* species both in our study and in previous reports [6–8, 13]. We identified one case of *A. sanguinis* and two cases of *A. urinale* bacteremia. However, MALDI-TOF MS was unable to reliably identify all isolates to the species level. Sequencing of the 16S rRNA gene proved to have a limited ability to separate *A. schaalii* from *A. sanguinis* and, therefore, some isolates could not be identified to the species level.

There are many similarities between *A. schaalii* and *Aerococcus urinae* [27]. Both species probably colonize the urinary tract and they have been difficult to correctly identify in the past. They cause invasive infections mostly in older males with underlying urogenital conditions, and are resistant to antibiotics commonly used to treat urinary infections. In fact, *Actinotignum* species and *Aerococcus* species have been isolated together from blood previously [18, 23] and, in our material, *Aerococcus* was the most common finding in polymicrobial *Actinotignum* bacteremia. Our previous description of *Aerococcus* bacteremia and the present description of *Actinotignum* bacteremia, however show some differences [23, 28, 29]. The mortality is higher in *Actinotignum* bacteremia as compared to *Aerococcus* bacteremia (16 vs. 8%) [18, 23]. This could either be due to specific bacterial virulence factors expressed by *Actinotignum* or also be due to host factors such as more advanced underlying conditions. Another difference is that *Actinotignum* species rarely seem to cause infective endocarditis as opposed to *Aerococcus* species, which cause endocarditis in a significant proportion of cases of bacteremia [23, 30, 31]. In our series, no case of endocarditis was found and only a single case of endocarditis caused by *Actinotignum* has been reported previously [9].

Only in one of the patients was *A. schaalii* isolated from urine. This is most likely due to routine practices in the clinical microbiology laboratory, which are unfavorable for the detection of *Actinotignum* species. Our findings, however, suggest that the urinary tract was the focus of the bacteremia in a majority of the cases. Many patients had underlying urological conditions and presented with signs and symptoms from the urinary tract. The patients with polymicrobial bacteremia had other typical uropathogens in their blood and urine cultures, such as *Aerococcus* species, *E. coli*, and *E. faecalis*, which also suggest a urinary tract focus. The patients who received a clinical diagnosis of pneumonia did not fulfill criteria necessary for that diagnosis and most of them likely had a urinary tract focus of infection.

There are no clinical break points established for *Actinotignum* species. However, from our results on antimicrobial susceptibility testing, β-lactams and vancomycin seem like feasible treatment options. Trimethoprim and ciprofloxacin display high MIC values and should be avoided. Previous studies have reported relapse of *A. schaalii* infections, which has led to suggestions of prolonged treatment [6, 19]. The median length of antibiotic treatment in this series was 2 weeks and, to our knowledge, there were no treatment failures. One patient had two episodes of *A. schaalii* bacteremia during a 6-month period, and this represented a recurrent infection rather than a treatment failure.

In conclusion, our results demonstrate that *Actinotignum* bacteremia is more common than previously thought and that it represents a condition with a significant fatality in elderly patients with underlying conditions. The urinary tract should be suspected as the primary focus of infection but, unfortunately, *Actinotignum* species seldom grow in ordinary urine culture conditions.
